# Impaired resolution of inflammatory response in the lungs of JF1/Msf mice following carbon nanoparticle instillation

**DOI:** 10.1186/1465-9921-12-94

**Published:** 2011-07-15

**Authors:** Koustav Ganguly, Swapna Upadhyay, Martin Irmler, Shinji Takenaka, Katrin Pukelsheim, Johannes Beckers, Martin Hrabé De Angelis, Eckard Hamelmann, Tobias Stoeger, Holger Schulz

**Affiliations:** 1 Comprehensive Pneumology Center, Institute of Lung Biology and Disease, Helmholtz Zentrum München, German Research Center for Environmental Health, Neuherberg/Munich, Germany; 2 Institute of Experimental Genetics, Helmholtz Zentrum München, German Research Center for Environmental Health, Neuherberg/Munich, Germany; 3 Center of Life and Food Sciences, Technical University Munich, Freising, Germany; 4 Department of Pediatric Pneumology and Immunology, Charité Universitätsmedizin Berlin, Berlin, Germany; 5 Department of Pediatrics, Ruhr-University Bochum, Bochum, Germany; 6 Department of Environmental and Occupational Health, University of Pittsburgh, Pittsburgh, PA, USA; 7 Institute of Epidemiology, Helmholtz Zentrum München, German Research Center for Environmental Health, Neuherberg/Munich, Germany

## Abstract

**Background:**

Declined lung function is a risk factor for particulate matter associated respiratory diseases like asthma and chronic obstructive pulmonary disease (COPD). Carbon nanoparticles (CNP) are a prominent component of outdoor air pollution that causes pulmonary toxicity mainly through inflammation. Recently we demonstrated that mice (C3H/HeJ) with higher than normal pulmonary function resolved the elicited pulmonary inflammation following CNP exposure through activation of defense and homeostasis maintenance pathways. To test whether CNP-induced inflammation is affected by declined lung function, we exposed JF1/Msf (JF1) mice with lower than normal pulmonary function to CNP and studied the pulmonary inflammation and its resolution.

**Methods:**

5 μg, 20 μg and 50 μg CNP (Printex 90) were intratracheally instilled in JF1 mice to determine the dose response and the time course of inflammation over 7 days (20 μg dosage). Inflammation was assessed using histology, bronchoalveolar lavage (BAL) analysis and by a panel of 62 protein markers.

**Results:**

24 h after instillation, 20 μg and 50 μg CNP caused a 25 fold and 19 fold increased polymorphonuclear leucocytes (PMN) respectively while the 5 μg represented the 'no observable adverse effect level' as reflected by PMN influx (9.7 × 10E3 vs 8.9 × 10E3), and BAL/lung concentrations of pro-inflammatory cytokines. Time course assessment of the inflammatory response revealed that compared to day1 the elevated BAL PMN counts (246.4 × 10E3) were significantly decreased at day 3 (72.9 × 10E3) and day 7 (48.5 × 10E3) but did not reach baseline levels indicating slow PMN resolution kinetics. Strikingly on day 7 the number of macrophages doubled (455.0 × 10E3 vs 204.7 × 10E3) and lymphocytes were 7-fold induced (80.6 × 10E3 vs 11.2 × 10E3) compared to day1. At day 7 elevated levels of IL1B, TNF, IL4, MDC/CCL22, FVII, and vWF were detected in JF1 lungs which can be associated to macrophage and lymphocyte activation.

**Conclusion:**

This explorative study indicates that JF1 mice with impaired pulmonary function also exhibits delayed resolution of particle mediated lung inflammation as evident from elevated PMN and accumulation of macrophages and lymphocytes on day7. It is plausible that elevated levels of IL1B, IL4, TNF, CCL22/MDC, FVII and vWF counteract defense and homeostatic pathways thereby driving this phenomenon.

## Introduction

A major component of urban air pollution is particulate matter (PM). Various epidemiological and clinical studies have shown the correlation between ambient PM concentration and adverse respiratory health effects throughout the developing countries and the industrialized world. Exposure to PM has been associated with an increased risk of various respiratory and cardiopulmonary diseases, increased mortality, and emergency room visits due to respiratory problems and restricted lung function [1-3]. Interestingly, several studies show that individuals with poor pulmonary function are expected to be at higher risk to respiratory diseases [4-6] like asthma or chronic obstructive pulmonary disease (COPD). Carbon black is an ingredient in rubber, plastics, inks and paints with an annual production of 10 million tons [7] indicating its wide usage and potentially massive exposure in day to day life among people of various working class. Carbon nanoparticles (CNP) also constitutes the core of combustion derived particles [8] and represents relevant surrogates for exhaust particles from modern diesel engines [9,10].

Inflammatory responses, triggered by pro-oxidative particle properties, are considered to attribute significantly to chronic pulmonary disease processes, such as COPD. Beside exposure to cigarette smoke, traffic and domestic heating as well as indoor house cooking are major sources of local combustion related particle exposures [11,12]. In this context ultrafine carbon particles are an important component of air pollution with respect to particle number and surface area. An increasing use of engineered nanoparticles in all spheres of life makes CNP also an evolving source of human exposure [13]. CNPs regardless of their different sources exhibit properties not displayed by their macroscopic counterparts. The high pulmonary deposition efficiency along with their large specific surface area, ready to interact with biological material and their potential to evade lung clearance by entering pulmonary cells is considered to be important in driving the emerging health effects of CNP linked to respiratory toxicity [14,15].

Previously C3H/HeJ (C3) and JF1/Msf (JF1) were detected to be the most divergent inbred mouse strains based on a pulmonary function screen [16-18]. For example, JF1 mice have smaller and less compliant lungs with larger conducting airway volumes compared to C3 mice. Therefore to approach experimentally the epidemiological finding of higher susceptibility for respiratory diseases among individuals with lower basal pulmonary function we have selected JF1, a strain previously characterized for limited pulmonary function as the physiological base [16-18] and physically-chemically well characterized, endotoxin free moderately toxic carbon nanoparticles (Printex 90) as the toxicological base in this study [19,20]. Through this broad explorative study our prime aim was to identify the potentially important molecular events taking place during the acute inflammatory response and its resolution following CNP exposure in JF1 mice and to compare the response to that of recently assessed in C3 mice, a strain with higher basal pulmonary function [21].

## Methods

The experiments were carried out using identical methodology as previously described by Ganguly et al [21].

### Particles

For CNP instillation endotoxin free Printex 90 particles obtained from Degussa (Frankfurt, Germany) were used as described earlier [21]. The primary particle size of Printex 90 is 14 nm, the specific surface area about 300 m^2^/g, and the organic content low, 1-2% [20]. Vials of 5 μg, 20 μg and 50 μg CNP particles in 50 μl were prepared just before use by suspending in pyrogen-free distilled water (Braun, Germany). The suspension of particles was sonicated on ice for 1 min prior to instillation, using a SonoPlus HD70 (Bachofer, Berlin, Germany) at a moderate energy of 20 Watt. We favor the use of distilled water for suspension of particles because the salt content of phosphate-buffered saline (PBS) causes rapid particle aggregation comparable to the "salting-out" effect and thus eliminates consistent instillation conditions. Particle characteristics have been described previously [21]. Briefly, The Zeta potential and intensity weighted median dynamic light scattering diameter of the printex 90 particles in a pyrogen free distilled water suspension at a concentration of 20 μg/50 μl using Zetatrac (Model NPA151--31A; Particle Metrix GmbH, Meerbusch, Germany) was 33 mV and 0.17 μm respectively.

### Mouse procedures

#### Animals

This study was approved by the Bavarian Animal Research Authority (Reference No: 55.2-1-54-2531-115-05). Female JF1/Msf (JF1) animals were purchased from the Jackson Laboratories (Bar Harbour, ME USA) at the age of 8 weeks. The animals were housed and acclimatized at the animal facility of Helmholtz Zentrum München under specific pathogen free conditions according to the European Laboratory Animal Science Association Guidelines [22] for at least 4 weeks. Food and water were available *ad libitum*. The experiments were performed with 12-14 weeks old animals. Mean body weight was 14.3 ± 1.7 g (mean ± SEM). Experimental groups were age matched and the age of 12-14 weeks was considered for this study so as to exclude the effect of any lung developmental events that may interfere with susceptibility. By the age of 10 weeks lung development is completed in mice and the lung is fully grown and has a mature structure [23].

Mice were anesthetized by intraperitoneal injection of a mixture of xylazine (4.1 mg/kg body weight) and ketamine (188.3 mg/kg body weight). The animals were then intubated by a nonsurgical technique [24]. Using a bulbheaded cannula inserted 10 mm into the trachea, a suspension containing 5, 20, or 50 μg CNP (Printex90) particles, respectively, in 50 μl pyrogene-free distilled water was instilled, followed by 100 μl air. Animals were treated humanely and with regard for alleviation of suffering.

#### Experimental design

Seven experimental groups were selected which included cage control, sham (vehicle) exposed, and CNP exposed (5 μg/day1, 20 μg/day1, 20 μg/day3, 20 μg/day7, 50 μg/day1) by intratracheal (i.t.) instillation. Cage control animals were not instilled, and sham animals received 50 μl pure distilled water (vehicle). The animal groups were designed so as to obtain an acute dose-response relationship [5 μg/day1, 20 μg/day1 and 50 μg/day1] and also to get a time course response [20 μg/day1, 20 μg/day3, 20 μg/day7] following i.t. instillation. Therefore 5 groups were exposed to particles and 2 groups served as control (cage control and sham exposed). Each of the seven experimental groups consisted of 11 animals (7 for lavage and 4 for histopathology) based on our previous experiences [21]. Out of the 7 lavaged animals, tissue samples from 4 mice were collected for protein analysis and 3 for future RNA studies. Four non-lavaged animals were used for histopathology. Lavaged lungs were immediately frozen in liquid nitrogen following dissection and stored at -80°C until next procedures for molecular analysis.

#### Bronchoalveolar lavage (BAL) and analysis

On day1/day3/day7 (as per experimental design) after instillation, mice were anesthetized by intraperitoneal injection of a mixture of xylazine and ketamine and sacrificed by exsanguination. BAL was performed accordingly (i.e. day1/day3/day7 after instillation) by cannulating the trachea and infusing the lungs 10 times with 1.0 ml PBS without calcium and magnesium, as described previously [21]. The BAL fluid from lavages 1 and 2 were pooled and centrifuged (425 *g*, 20 min at room temperature). The cell-free supernatant from lavages 1 and 2 were pooled and stored at -20°C immediately for biochemical measurements such as total protein and panel assays. The cell pellet from lavages 1 and 2 was resuspended immediately in 1 mL RPMI 1640 medium (BioChrome, Berlin, Germany) and supplemented with 10% fetal calf serum (Seromed, Berlin, Germany); the number of living cells was determined by the trypan blue exclusion method. We performed cell differentials on the cytocentrifuge preparations (May-Grünwald- Giemsa staining; 2 × 200 cells counted). We used the number of polymorphonuclear leukocytes (PMNs) as a marker of inflammation. Total protein content was determined spectrophotometrically at 620 nm, applying the Bio-Rad Protein Assay Dye Reagent (no. 500-0006; BioRad, Munich, Germany). We analyzed 50 μl BAL/mouse for panel assays.

#### Histology

Four not lavaged animals per experimental group were used for histological analysis. Mice were sacrificed by an overdose of ketamin and the lungs were inflation-fixed at a pressure of 20 cm H_2_O by instillation of phosphate buffered 4% formaldehyde. Three cross slices of the left lobe and 4 slices of each right lobe were systematically selected and embedded in paraffin, and 4 μm thick sections were stained with hematoxylin and eosin. The sections were then studied by light microscopy.

#### Protein panel assays

In this study we analyzed the identical set of 62 protein markers as already introduced while studying CNP exposure in C3 mice [21]. A detailed list of each of the 62 markers, their gene symbol and associated gene ontology terms, least detectable dose (LDD) etc. is supplied in Additional File 1, Table S1. Our panel of markers are known to play important roles in the following key processes of lung tissue: **i) **Initiation and amplification of inflammation **ii) **Induction of T-cell independent macrophage activation **iii) **Regulation of dendritic cell maturation and differentiation, and **iv) **Regulation of T-cell activation and differentiation as described by [25].

Total lung homogenate was prepared using 50 mM Tris-HCL with 2 mM EDTA, pH 7.4 as the lysis buffer (1000 μl) from 4 animals/experimental group using the whole lung. Using the Rodent MAP™ version 2.0 of the Rules Based Medicine (Austin, Texas) a panel of mostly proinflammatory and inflammatory markers was analyzed from total lung homogenate and BAL. BAL and lung homogenates were always taken from the same animals to avoid any inter-animal variation. BAL of the 4 animals/group was pooled for the measurement and only the markers equal to/above (≥) the sensitivity level were considered. BAL was pooled from 4 animals as our focus was on the lung homogenate considering BAL concentrations of proteins are often below LDD. Sensitivity level was the LDD as provided by Rules Based Medicine. We considered in pooled samples the markers below sensitivity levels to be not reliable due to lack of scope for reproducibility in multiple independent samples. However, in the lung homogenate markers below LDD were also considered for analysis and discussion as we could measure samples from 4 independent animals/group. In most cases little variance between replicates was observed.

Additionally three more markers hemoxygenase-1 (HO-1; Stressgen Catalog # 960-071), osteopontin (SPP1; Stressgen Catalog # 900-090A) and lipocallin-2 (LCN2; R&D Systems Catalog # DY1857) were assayed from the same samples using the respective ELISA kits.

#### Heatmaps and pathway analysis

Protein expression data from lung tissue (means, n = 4) and BALF (pools from 4 animals) were used for heatmap generation. Protein concentrations were normalized to the highest value for each protein (set to equal 1) and the resulting values were used as input for heatmap generation with CARMAweb [26]. The Ingenuity Pathway Analysis tool was used to generate the interaction network for selected regulated proteins and to identify their biological functions.

#### Statistics

A two-way analysis of variance (ANOVA) was used to analyze differences between control and various exposure groups. P values less than 0.05 were considered as statistically significant. All computations were done by the software packages Statgraphics plus v5.0 (Manugistics, Rockville, MD) and SAS V9.1 (Cary, NC). All the data were normally distributed (F-test). Data are presented as arithmetic mean values of n observations ± the standard error (SE).

## Results

The exposure groups were designed to assess an acute dose-response relationship one day after intratracheal instillation of 5 μg (0.35 g/kg BW), 20 μg (1.4 g/kg BW) and 50 μg Printex 90 (3.5 g/kg BW). The time course response was obtained for the moderate dose of 20 μg/mouse lung on day 1, day 3 and day 7. We have not observed any significant difference between cage control and sham exposed control animals in any of the measurements performed using BAL and lung homogenate.

### Dose and time response of BAL cells

The total number of retrieved BAL leucocytes was not affected after i.t. instillation of 5 μg CNP (0.27 ± 0.04 × 10E6 cells/lung versus 0.27 ± 0.05 × 10E6 cells/lung in sham exposed mice) but increased two fold to 0.53 ± 0.06 × 10E6 cells/lung at day 1 (p < 0.05) after instillation of 20 μg CNP (data not shown). The time course analysis revealed an intermittent decline of BAL leucocytes almost to control level on day 3 (0.31 ± 0.07 × 10E6 cells/lung, n.s.) followed by an increase to 0.51 ± 0.11 × 10E6 cells/lung on day 7, i.e. to 188% of that observed in sham exposed mice.

### PMN

**Figure 1:**
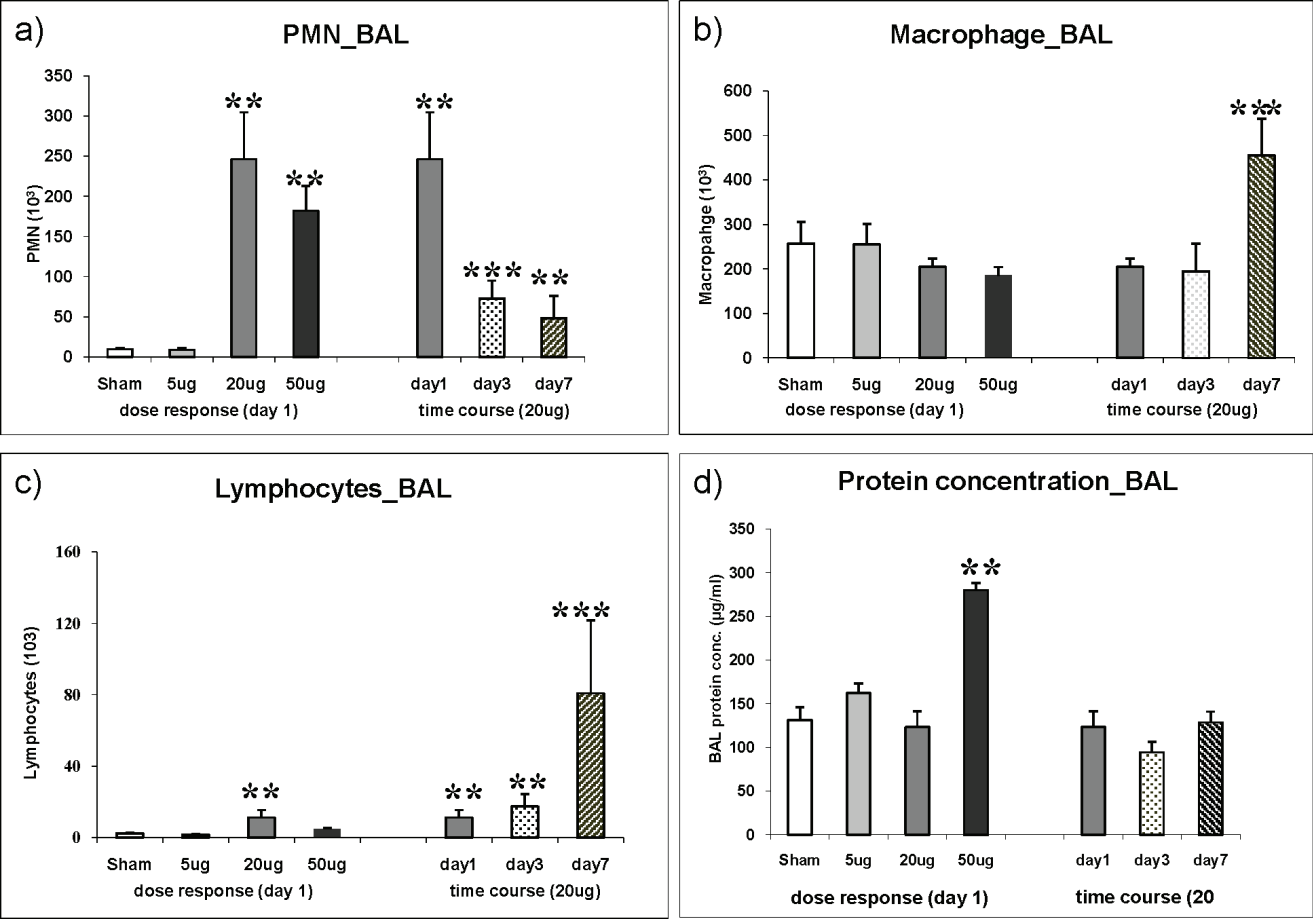
**Bronchoalveolar lavage fluid (BAL) cell differentials and protein concentration**. Dose dependent influx and time dependent resolution of (a) polymorphonuclear leukocytes (PMN), (b) macrophages, and (c) lymphocytes in the BAL following intratracheal (i.t.) instillation of carbon nanoparticles particles in the JF1/Msf (JF1) mice (n = 7 animals/experimental group). Total protein concentration is provided in Figure 1d. (**) Significantly different with respect to (w.r.t) both sham control and 5 μg exposed; (***) significantly different w.r.t 20 μg exposed at day1, sham control and 5 μg exposed; p ≤ 0.05.

As observed for the total cell count no significant induction of PMN was detected following i.t instillation of 5 μg CNP (8.9 ± 2.3 × 10E3 PMNs) compared to control (9.7 ± 1.5 × 10E3 PMNs, Figure 1a). The increase of PMN numbers detected on day1 after 20 μg or 50 μg CNP instillation already reached saturation at the 20 μg dosage. A 25-fold induction of PMN was detected following 20 μg (246 ± 58 × 10E3 PMNs) and a 19-fold after 50 μg (182 ± 31 × 10E3 PMNs) CNP instillation. Time course analysis of PMN numbers revealed significantly reduced PMN counts after 3 days (73.9 ± 22.1 × 10E3 PMNs) and 7 days (48.5 ± 27.4 × 10E3 PMNs) compared to that of day1. However at day 3 and day 7 the PMN count remained 8 and 5 times higher (< 0.05), respectively, than the baseline values indicating incomplete resolution of the neutrophil influx related inflammation following 20 μg of CNP instillation.

### Macrophages

No dose dependent increase of macrophage numbers was observed (Figure 1b). Strikingly, in the time course analysis an obvious, 1.8-fold induction of macrophage numbers was detected at day 7 (455 ± 83 × 10E3, vs. sham 257 ± 48 × 10E3, p < 0.05).

### Lymphocytes

The acute response of lymphocytes on day 1 is to some extent comparable to that of PMNs showing the highest influx with the 20 μg but not at the 50 μg dosage (Figure 1c). Strikingly, in the time course analysis a moderate induction of lymphocyte numbers in response to 20 μg CNP was detected on day 1 (4.9 times) and day3 (7.6 times) followed by a strong induction of lymphocyte numbers being detected on day 7 (35.1 times). It is interesting to note that the time course response of lymphocytes resembles that of the macrophages as both cell types show a maximum influx at day 7.

### BAL protein concentration

Compared to sham exposed mice, only the instillation of the highest dose (50 μg/mouse) caused a significant (2.1 fold) increase in total BAL protein concentration (131.5 ± 14.5 μg/ml versus 280.1 ± 8.3 μg/ml, p < 0.05) 1 day after CNP instillation indicating alveolar barrier injury with capillary leakage only at this concentration (Figure 1d). Time course investigation of 20 μg instilled lungs revealed no changes of BAL protein concentrations from day 1 to day 3 and day 7.

### Histopathology

**Figure 2:**
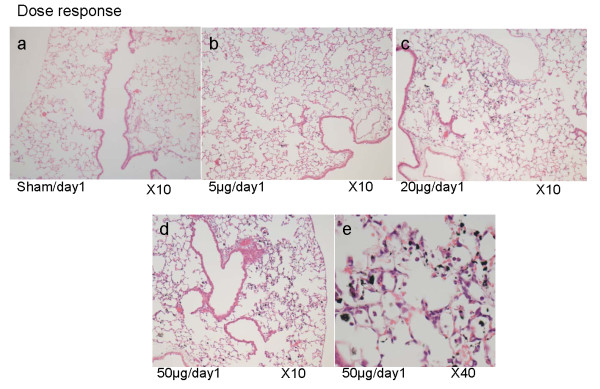
**Dose dependent accumulation of carbon nanoparticle laden macrophages in the lung**. Histopathological analysis of the paraffin embedded JF1 lung sections (n = 4) showed a dose dependent accumulation of carbon nanoparticle laden macrophages (2a-e). (a) Sham/day1, (b) 5 μg/day1, (c) 20 μg/day1, (d,e) 50 μg/day1; (magnification: 2a-d:10×, 2e:40×).

Histopathological analysis of paraffin embedded JF1 lung sections (n = 4) showed a typical dose dependent accumulation of particle laden macrophages on days 1, 3 and 7 (Figure 2a-e). In 50 μg/day1 samples inflammatory cell infiltration (PMN) was clearly visible (Figure 2e) whereas at 20 μg/day1 only slight PMN infiltration was detectable (data not shown).

### Molecular analysis for lung and BAL compartment

In the present study a panel of 62 protein markers was applied to assess the CNP response in JF1 mice as previously described for C3 mice [21]. A detailed list of each of the 62 markers is supplied in Additional File 1, Table S1.

### Lung compartment

**Figure 3:**
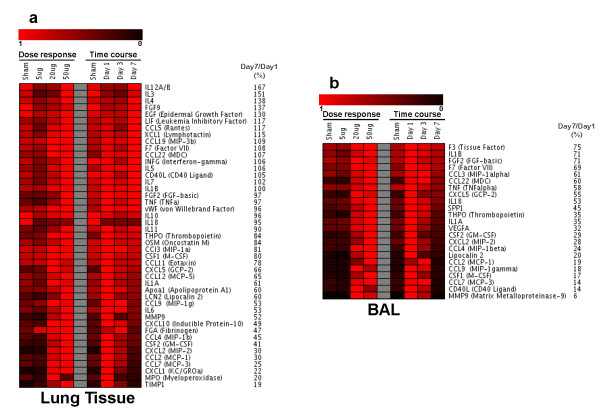
**Heatmap representation of protein concentration levels in lung homogenate and bronchoalveolar lavage (BAL) following intratracheal instillation of carbon nanoparticles in JF1/Msf mice**. Shown are normalized data (highest value is set to "1") for dose or time response. Red color indicates increasing concentrations. Protein symbols and relative expression day7/day1 (in %) are indicated.

From the 62 markers 17 proteins did not exhibit any response in lung and were therefore not considered for analysis (CD40, CRP, EDN1, FGF9, F3, haptoglobin, IgA, IL17, IL2, SAP, SCF, SGOT, TF, HO-1, GST-α, myoglobin, VCAM1). Dose and time course responses of the remaining 45 proteins are represented as a heat map in Figure 3. In the CNP dose response most proteins showed a strong upregulation already at a dose of 20 μg and the expression level of 14 of them did not significantly increase further at a dose of 50 μg (F7, FGA, GCP2/CXCL5, MCP1/CCL2, MCP3/CCL7, MCP5/CCL12, IP10/CXCL10, KC/CXCL1, MDC/CCL22, MIP1b/CCL4, MIP2/CXCL2, MIP1g/CCL9, THPO and vWF). This observation can be associated with the dose response of PMN numbers in the BAL as these proteins include the major PMN recruiters CXCL1, 2, 5 and 10. From the 45 proteins 13 proteins were not significantly regulated at 20 μg/day1 but were significantly induced at 50 μg/day1 (APOA1, CD40L, EGF, FGF2, IFN-gamma, IL18, IL1B, IL3, IL4, IL5, M-CSF/CSF1, MIP1α/CCL3, and RANTES/CCL5).

For the time course study the majority of proteins exhibited the expected response pattern of initial increase (day1) and decline to baseline levels by day 7 following 20 μg CNP exposure. Among them were: IL1B, MCP1/CCL2, MIP1/CCL4, MMP9, MCP3/CCL7, IP10/CXCL10, MPO, MIP2/CXCL2, IL6, GM-CSF/CSF2, MIP1-gamma/CCL9, GCP-2/CXCL5, TIMP1, FGA, MCP-5/CCL12, KC/CXCL1. As evident from the list, the major PMN recruiters CXCL1, 2, 5 and 10 were down regulated at day 7 compared to day 1 reflecting the phenotypic observation of an obvious decline in PMN numbers until day 7 (Figure 3).

**Figure 4:**
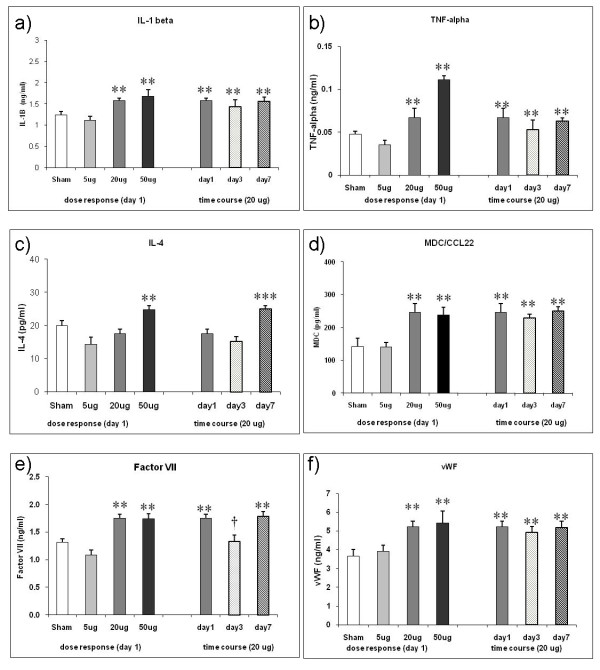
**Time course response markers in the lung homogenate following intratracheal (i.t.) instillation of carbon nanoparticles (CNP) in JF1/Msf (JF1) mice**. **(a-f) **Shown are markers with remaining elevated levels at day 7 after an initial significant induction at day1 (except IL4) in response to 20 μg i.t. instillation of CNP. F7: Factor VII; IL1B: Interleukin-1beta; IL4: Interleukin-4; MDC/CCL22: Macrophage-Derived Chemokine; TNF: Tumor Necrosis Factor-alpha; vWF: von Willebrand Factor; (**) Significantly different with respect to (w.r.t) both sham control and 5 μg dose; (†) significantly different w.r.t 20 μg exposed at day1; (***) significantly different w.r.t 20 μg at day1, 5 μg and sham control. p ≤ 0.05; (n = 4 animals/experimental group).

Further, we have detected 6 informative time course markers in the lung homogenate which showed an initial shoot up and remained at elevated levels until day 7 thereby correlating in particular with increased BAL cell differentials of alveolar macrophages and lymphocytes. These markers are IL1B, TNF, IL4, MDC/CCL22, F7, and vWF (all except vWF are at/above sensitivity level of detection). Their individual response pattern for the dose and the time response is shown in Figure 4. Interestingly IL4 in spite of not exhibiting an obvious dose response was detected at significantly higher levels at day 7 compared to sham, 5 μg and 20 μg/day1 and day 3. A detailed list of each of the 62 markers is provided in Additional File 1, Table S1.

### BAL compartment

For BAL, only markers detected at/above sensitivity level (see methods, n = 4 BAL samples were pooled/experimental group) were considered. This were 23 out of 62 markers as represented in the heatmap (Figure 3). With respect to the dose response, these 23 markers can be categorized in (i) BAL markers exhibiting increasing levels from 20 μg to 50 μg CNP/day1 being CCL22, SPP1, CSF2, CXCL2, Lipocalin 2/LCN2, CCL2, CSF1, CCL7 and THPO; (ii) BAL markers exhibiting saturation at 20 μg/day1, i.e. comparable levels at 20 μg and 50 μg/day1 being F3, IL1B, FGF2, F7, CCL3, CCL4, TNF, IL18, IL1A, VEGFA, and MMP9; and (iii) BAL markers (CXCL5, CCL9 and CD40L) exhibiting highest concentrations at 20 μg/day1 and lower ones at 50 μg dosage similar to lymphocyte numbers observed in BAL samples during the dose response study.

With respect to the time course, 19 of the 23 BAL markers reached their baseline levels by day7 in 20 μg samples. Four markers - CCL22, CXCL5, CCL9 and MMP9 -exhibit a clear decline in concentration from day 1 to day7 but were somewhat elevated (1.5-2.5 fold higher) than baseline level on day 7.

### Biological pathway analysis

**Figure 5:**
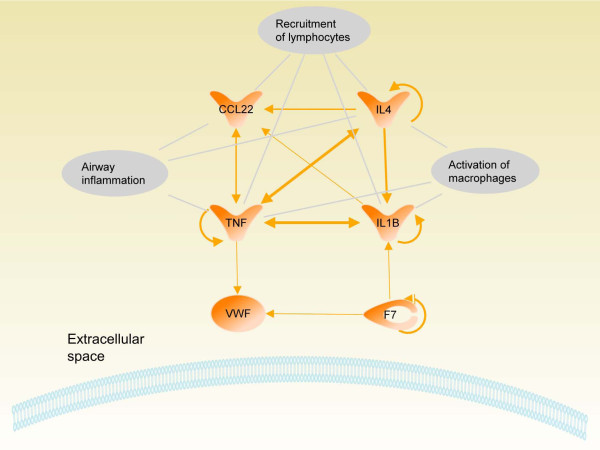
**Pathway analysis of proteins detected at elevated concentrations during time course analysis in lung following intratracheal instillation of 20 μg carbon nanoparticles (CNP)**. At day 7 when PMN levels were 5 times higher compared to baseline and a sudden influx of lymphocytes and macrophages was detected, Interleukin-1beta (IL1B), Tumor necrosis factor-alpha (TNF), Factor VII (F7), Interleukin-4 (IL4), Macrophage-derived chemokine (MDC/CCL22) and von Willebrand Factor (vWF) remained at significantly elevated concentrations compared to their baseline levels. The predicted molecular network indicates the role of these markers in airway inflammation, recruitment of lymphocytes and activation of macrophages. Shown are the extracellular space, part of the cell membrane and published interactions are indicated by arrows (line thickness represents the number of references).

We identified 6 markers (IL1B, TNF, IL4, MDC/CCL22, F7, and vWF) which remained in elevated concentrations in the lung compartment (i.e. concentrations on day 7 were significantly higher to both sham and 5 μg but not significantly lower to 20 μg/24 h or 20 μg/72 h). This can be associated with the delayed macrophage- and lymphocyte influx. Based on these findings we performed an extensive literature study which revealed a close interaction between these markers which was also observed by an analysis with the Ingenuity pathway software. Known functions associated with the six marker proteins are airway inflammation, activation of macrophages and recruitment of lymphocytes (Figure 5).

## Discussion

Previously we observed large variation in pulmonary function among inbred mouse strains [17,18,27] and found that C3H/HeJ (C3) and JF1/Msf (JF1) to be the most divergent strains. In comparison to C3, JF1 mice have smaller, less compliant lungs with large conducting airway volumes and distinct morphological differences, like small alveolar dimensions. In JF1, total lung capacity (TLC, female) is 60% to that of C3, the static lung compliance is only less than half to that in C3, and the contribution of airway volume to TLC is 1.5 times more in JF1 than C3 [18]. The mean alveolar chord length is also smaller in JF1 compared to C3 [28]. Alveolar surface area being the site of immediate interaction is however similar among JF1 and C3 [28] (Table 1). Since lung size in mice shows only a moderate correlation to body weight [18] and the alveolar surface area does not differ among C3 and JF1 we decided to use the same CNP dosage for both strains to assess their acute inflammatory response and its resolution.

**Table 1:** Comparison of lung characteristics between female C3H/HeJ and JF1/Msf mice.

Parameter	C3H/HeJ	JF1/Msf	Reference
Body weight (g)	22.4 ± 0.6	16.5 ± 0.4	[18]

Total Lung Capacity (TLC)(μl)	1443 ± 29	874 ± 17	[17,18]

Static Lung Compliance (sC_L_) (cmH_2_O/ml)	84.8 ± 3.6	35.5 ± 2.2	[17,18]

Dead Space (V_D_) volume (%)	15.8 ± 0.3	22.1 ± 0.5	[17,18]

Mean alveolar Chord length (μm)	69.2 ± 14.8	48.5 ± 5.5	[28]

Alveolar surface area (cm^2^)	847 ± 70	906 ± 102	[28]

### Pulmonary response to CNP in JF1 mice

The dose response of JF1 to CNP exposure was characterized by a marked PMN cell influx (up to 25-fold induction) at day 1 reaching a saturation level already after instillation of 20 μg CNP whereas at that time point no considerable macrophage recruitment into the alveolar space occurred. The marked influx of PMNs was associated with a number of elevated inflammatory cytokine levels, such as CXCL1, 2, 5, 10, CSF2, IL1B, CCL2, CCL7, TNF in lung tissue and partly also in BAL fluid (Figure 3). These cytokines are well known to be involved in PMN recruitment, [29]. Also extracellular matrix protecting proteins (TIMP1) and degrading enzymes (MMP9) were considerably induced. Typically, the acute inflammatory response to CNP is characterized by recruitment of PMN to the alveolar space with resolution and normalization of cell numbers after 3 to 7 days. However, detection of 5 times higher than baseline levels of PMN on day 7 after CNP exposure indicates that the ongoing resolution process is not complete at this time point in JF1 mice. The impaired resolution of inflammation is further supported by the delayed influx of macrophages (2-fold increased) and considerable recruitment of lymphocytes (35-times induced) into the airway lumen one week after treated with 20 μg CNP. From the panel of protein markers analysed we have detected 6 markers - IL1B, IL4, MDC/CCL22, TNF, F7, and vWF - which remained elevated up to day 7. Our pathway analysis revealed a close interaction between these markers (Figure 5) and their implication in airway inflammation, activation of macrophages and recruitment of lymphocytes.

The proinflammatory markers TNF and IL1B remained elevated between day 1 and day 7. Elevated levels of IL1B and TNF in lung tissue are consistent with the observed impaired clearance of PMN from the lungs [30] resulting in the higher than baseline PMN levels on day 7. Due to their pleotrophic proinflammatory properties it is plausible that they are also involved in elevated numbers of alveolar macrophages detectable at day 7. In this context it is interesting to note that the cytokine IL4 exhibited increased levels either at high dosage (50 μg CNP) on day 1 or at the 20 μg dose on day 7. IL-4 is considered to be involved in chronic inflammatory responses [31] and is deeply involved in allergic airway inflammation, in regulation of lymphocyte differentiation of naïve helper T-cells to Th2 cells [32] as well as modulating the TNF mediated growth and differentiation of dendritic cells (DC) [33,34]. IL4 favors the polarisation of macrophages to the M2a type [31]. The macrophage derived chemokine CCL22 is a CC chemokine produced in a constitutive way by M2 macrophages [31], but also by dendritic cells (DC) and by activated B lymphocytes. CCL22 is chemotactic for DC, natural killer cells and activated T lymphocytes [35-37]. Taken together, our study shows that JF1 exhibit an impaired resolution of inflammation in response to CNP exposure. The delayed clearance of many pro-inflammatory cytokines which peaked at day 1 after CNP exposure together with still increased levels of IL1B, TNF, IL4, CCL22 could be associated with the delayed clearance of PMNs and increased numbers of macrophage and lymphocytes in the JF1 lungs detected one week after CNP exposure.

### Comparison of pulmonary responses to CNP in C3 and JF1 mice

C3 mice were previously subjected to an identical exposure protocol as JF1 mice [21]. Although C3 mice showed a strong inflammatory response to CNP as evidenced by influx of PMNs into the alveolar space at the highest dose (~40 fold induction at 50 μg CNP) and the induction of most of the biomarkers at day 1 (49 out of 62 were upregulated) they were able to completely resolve the PMN influx within 7 days. Moreover, in C3 mice cell numbers of macrophages and lymphocytes were not affected (Table 2). A defense/homeostasis pathway was activated, with IL1B/IL18, EDN1, FGF2, and VEGF [21] as the major players. Table 2 illustrates the distinct different responses to CNP in C3 and JF1 mice for BAL cells and selected biomarkers being either elevated at day 7 in C3 or JF1 mice. However, in response to CNP, many of the inflammatory biomarkers which were initially upregulated and remained elevated in C3 were not affected in JF1, like IL18, VEGF, CCL3, CCL5, Lymphotactin/XCL1, F3, EDN, CRP, APCS. On the other hand, in JF1 levels of TNF, IL4, CCL22, F7, vWF remained elevated at day7 which were though initially upregulated in C3 but most of them reached baseline levels at day 7. In particular, in JF1 mice elevated levels of IL1B and TNF were detected but not IL18 which was involved in the induction of EDN1/FGF2/VEGF homeostatic pathway in C3. This may in part explain the deleterious effects of CNP in JF1 mice. SOD3 is the main extracellular antioxidant enzyme of the lung. JF1 mice have 2-3 fold lower superoxide dismutase 3 (*Sod3*) transcript, protein and also activity levels in the lungs compared to C3 [16,38]. Interestingly, Laurila at al. [39] showed that SOD3 significantly reduces inflammatory cell migration of mononuclear cells after ischemic damage by regulating adhesion molecules and cytokine expression, among them TNF, which stayed elevated in JF1. Hence, it is reasonable to assume that depletion of SOD3 can alter the response to particle mediated oxidative stress. It may be plausible that the difference in the levels of superoxide dismutase 3 in the lungs of between C3 and JF1 influence the different response pattern to CNP.

**Table 2:** Time response in JF1 and C3 mice after single instillation of 20 μg Printex 90: BAL cell differentials and protein markers expressed in lung tissue at day 1 and day 7

		JF1/Msf		C3H/HeJ	
		**Day1**	**Day7**	**Day1**	**Day7**

**BAL cell differentials**	**PMN**	**↑**	↑	**↑**	↑ ↔

	**Macrophages**	NR	↑	NR	NR

	**Lymphocytes**	NR	↑	NR	NR

					

**Lung tissue proteins**	**IL1B**	↑	↑	↑	↑

	**TNF**	↑	↑	↑	↔

	**IL4**	↔	↑	↑	↔

	**CCL22**	↑	↑	↑	↔

	**F7**	↑	↑	↑	↔

	**vWF**	↑	↑	↑	↑

	**IL18**	↓	↓	↑	↑

	**VEGF**	NR	NR	↑	↑

	**CSF1**	↑	↔	↑	↑

	**CCL11**	↑	↔	↑	↑

	**CCL5**	NR	NR	↑	↑

	**CCL9**	↑	↔	↑	↑

	**CD40L**	NR	NR	↑	↑

	**CCL4**	↑	↔	↑	↑

	**XCL1**	NR	NR	↑	↑

	**CXCL5**	↑	↑ ↔	↑	↑

	**CCL3**	NR	NR	↑	↑

	**F3**	NR	NR	↑	↑ ↔

	**FGF2**	NR	NR	↑	↑

	**EDN1**	NR	NR	↑	↑

	**APCS**	NR	NR	↑	↑

	**FGA**	↑	↔	↑	↑

	**THPO**	↑	↑ ↔	↑	↑

**↑**: Higher than control; **↓**: Lower than control; **↔**: Equivalent to control; **↑↔**: higher than control but lower than day 1; NR: no response; (C3H/HeJ data from reference 21)

## Conclusion

JF1 exhibit an impaired resolution of inflammation in response to CNP exposure evidenced by a delayed clearance of PMNs from the lungs and an influx of mononulclear cells into the lungs one week after exposure. At the molecular level, this phenotype was associated with elevated levels of IL1B, TNF, IL4, CCL22, and the lack of establishing the defense/homeostasis pathway as recently proposed for C3 mice [21] which involves the upregulation of IL18, EDN1, FGF2, and VEGF. This suggests an increased susceptibility of JF1 to extrinsic insults and a potential disposition to continuing of inflammatory processes. Therefore these explorative studies identified plausible molecular pathways causing the different response to CNP in JF1 and C3 mice. Further pathway directed investigations are required to improve our understanding on the genetic and molecular basis of CNP induced inflammatory response.

## Competing interests

The authors declare that they have no competing interests.

## Authors' contributions

KG, SU, EH, HS and TS conceived and designed the experiments. KG, SU, ST, KP performed the experiment; KG, SU, ST, MI, JB, HS and TS analyzed the data; KG, SU, MI, MHA, TS and HS wrote the manuscript. All authors read and approved the final manuscript. Drs. Schulz and Stoeger contributed equally to this article

## Supplementary Material

Additional file 1 **Table S1**. List of all analyzed proteins, their respective gene symbols, Entrez identification numbers, associated gene ontology terms according to the Mouse Genome Informatics (MGI) database, least detectable doses (LDD), and expression levels in BAL and lung. Click here for file
