# Supplementary material for: Impaired resolution of inflammatory response in the lungs of JF1/Msf mice following carbon nanoparticle instillation
Source: Respir Res. 2011 Jul 15;12(1):94. doi: 10.1186/1465-9921-12-94 (PMC3145576; doi:10.1186/1465-9921-12-94)
Supplement: Additional file 1 — Table S1. List of all analyzed proteins, their respective gene symbols, Entrez identification numbers, associated gene ontology terms according to the Mouse Genome Informatics (MGI) database, least detectable doses (LDD), and expression levels in BAL and lung. [file 1465-9921-12-94-S1.DOC]

**Supplementary Table S1**

List of all analyzed proteins, their respective gene symbols, Entrez identification numbers, associated gene ontology terms according to the Mouse Genome Informatics (MGI) database, least detectable doses (LDD), and expression levels in bronchoalveolar lavage (BAL) and lung. The column category (i-v) is based on key processes of lung tissue [24] : **1)** Initiation and amplification of inflammation **2)** Induction of T-cell independent macrophage activation **3)** Regulation of dendritic cell maturation and differentiation, **4)** Regulation of T-cell activation and differentiation and **5)** modify connective tissue structures and regulate blood vessel growth. Our panel of markers are known to be mainly within categories (i-iv) which we allocated based on general scientific knowledge.

| **Marker** | **Gene Symbol** | **Entrez Gene ID** | **Associated GO terms** | **Category** | **Least Detectable Dose (LDD)** | **Expression in Pooled BAL samples**  **≥LDD** | **Expression in Lung homogenate**  **≥LDD** |
| --- | --- | --- | --- | --- | --- | --- | --- |
| Apolipoprotein A1 | *Apoa1* | 11806 | Blood vessel endothelial cell migration; cholesterol biosynthetic process | **1** | 10 ug/mL | × | × |
| CD40 antigen | *Cd40* | 21939 | B-cell activation; Immune response (IR) | **4** | 12 pg/mL | × | √ |
| CD40 Ligand | *Cd40lg* | 21947 | B-cell differentiation, IR, Isotype switching | **4** | 92pg/mL | √ | √ |
| C Reactive Protein (CRP) | *Crp* | 12944 | Acute phase response | **1** | 0,83ug/mL | × | × |
| Epidermal Growth Factor (EGF) | *Egf* | 13645 | MAPKKK cascade; branching morphogenesis | **1** | 39pg/mL | × | × |
| Endothelin-1 | *Edn1* | 13614 | Blood vessel morphogenesis | **1 (5)** | 67pg/mL | × | × |
| Eotaxin | [*Ccl11*](http://www.ncbi.nlm.nih.gov/sites/entrez?Db=gene&Cmd=ShowDetailView&TermToSearch=20292&ordinalpos=1&itool=EntrezSystem2.PEntrez.Gene.Gene_ResultsPanel.Gene_RVDocSum) | 20292 | Chemotaxis (C); IR; Inflammatory response (Inf R) | **1** | 12pg/mL | × | √ |
| Factor VII | *F7* | 14068 | Blood coagulation | **1 (5)** | 0,96ng/mL | √ | √ |
| Fibroblast Growth Factor-9 (FGF-9) | *Fgf9* | 14180 | MAPKKK cascade; Lung development | **1 (5)** | 0,99ng/mL | × | √ |
| Fibrinogen | *Fga,Fgb,Fgg* | 14161,110135,99571 | Blood coagulation | **1 (5)** | 170ug/mL | × | √ |
| Granulocyte Chemotactic Protein-2  (GCP-2 ) | *Cxcl5* | 20311 | C; IR; Inf R | **1** | 0,025ng/mL | √ | √ |
| Granulocyte Macrophage-Colony Stimulating Factor  (GM-CSF ) | *Csf2* | 12981 | IR | **1, 3, 4** | 8,7pg/mL | √ | √ |
| Glutathione S-Transferase alpha  (GST-α ) | *Gsta1-4* | 14857-14860 | ROS; NOS | **1** | 0,42ng/mL | Not detectable | Not detectable |
| Haptoglobin | *Hp* | 15439 | [Proteolysis](http://www.informatics.jax.org/searches/GO.cgi?id=GO:0006508) | **1** | 0,64ug/mL | √ | √ |
| Interferon-gamma  (IFN-γ) | *Ifng* | 15978 | IR; positive regulation of T-cell proliferation; Inflammatory cell apoptosis; Neutrophil apoptosis; Neutrophil chemotaxis | **2, 4** | 68pg/mL | × | × |
| IgA | *Igh-2* | 238447 | Systemic | **1** | 1,9ug/mL | × | × |
| Interleukin-10 | *Il10* | 16153 | IR; negative regulation of B-cell proliferation; negative regulation of IL-12 production | **2, 4** | 109pg/mL | √ | √ |
| Interleukin-11 | *Il11* | 16156 | MAPKKK cascade | **1** | 87pg/mL | × | × |
| Interleukin-12p70 | *Il12b* | 16159, 1616 | Positive regulation of T-cell proliferation | **2, 4** | 0,57ng/mL | × | × |
| Interleukin-17 | *Il17a* | 16171 | Inf R | **1, 4** | 0,15ng/mL | × | √ |
| Interleukin-18 | *Il18* | 16173 | IR; Angiogenesis | **4** | 0,67ng/mL | √ | √ |
| Interleukin-1alpha | *Il1a* | 16175 | IR; Inf R; Angiogenesis | **1** | 45pg/mL | √ | √ |
| Interleukin-1beta | *Il1b* | 16176 | Fever; Inf R; IR; VEGF production (positive regulation); JNK cascade; Leucocyte migration; Neutrophil chemotaxis | **1** | 0,45ng/mL | √ | √ |
| Interleukin-2 | *Il2* | 16183 | IR; Negative regulation of Inf R; Negative regulation of lymphocyte proliferation; positive regulation of T-cell proliferation | **4** | 67pg/mL | × | × |
| Interleukin-3 | *Il3* | 16187 | IR; JNK cascade | **3** | 21pg/mL | × | √ |
| Interleukin-4 | *Il4* | 16189 | B-cell activation; IR; cholesterol metabolic process | **3, 4** | 74pg/mL | × | √ |
| Interleukin-5 | *Il5* | 16191 | IR | **4** | 0,19ng/mL | × | √ |
| Interleukin-6 | *Il6* | 16193 | Acute phase response; IR; MAPKKK cascade; Neutrophil apoptosis | **1** | 14pg/mL | × | √ |
| Interleukin-7 | *Il7* | 16196 | Anti-apoptosis; IR; positive regulation of B-cell proliferation; positive regulation of T-cell proliferation | **4** | 0,31ng/mL | × | × |
| Inducible Protein-10  (IP-10 ) | *Cxcl10* | 15945 | C; IR; Inf R | **1** | 40pg/mL | × | √ |
| Melanoma Growth Stimulatory Activity Protein  (KC/GROalpha ) | *Cxcl1* | 14825 | IR; Inf R | **1** | 0,17ng/mL | × | √ |
| Leukemia Inhibitory Factor  (LIF) | *Lif* | 16878 | IR; MAPKKK; Stem cell maintenance; Macrophage differentiation | **1** | 44pg/mL | × | √ |
| Lymphotactin | *Xcl1* | 16963 | C; IR | **4** | 85pg/mL | × | √ |
| Monocyte Chemoattractant Protein-1  (MCP-1) | *Ccl2* | 20296 | C; IR; Inf R | **1, 4** | 17pg/mL | √ | √ |
| Monocyte Chemoattractant Protein-3  (MCP-3) | *Ccl7* | 20306 | C; IR; Inf R | **1, 4** | 31pg/mL | √ | √ |
| Monocyte Chemoattractant Protein-5  (MCP-5) | *Ccl12* | 20293 | C; IR; Inf R | **1, 4** | 46pg/mL | × | √ |
| Macrophage-Colony Stimulating Factor  (MCSF) | *Csf1* | 12977 | Macrophage differentiation; Monocyte differentiation | **2** | 0,018ng/mL | √ | √ |
| Macrophage-Derived Chemokine  (MDC) | *Ccl22* | 20299 | C; IR; Inf R | **2** | 22pg/mL | √ | √ |
| Macrophage Inflammatory Protein-1alpha  (MIP-1α) | *Ccl3* | 20302 | C; IR; Inf R | **1** | 0,23ng/mL | √ | √ |
| Macrophage Inflammatory Protein-1beta  (MIP-1β) | *Ccl4* | 20303 | C; IR; Inf R | **3, 4** | 78pg/mL | √ | √ |
| Macrophage Inflammatory Protein-1gamma  (MIP-1γ) | *Ccl9* | 20308 | C; IR | **3** | 0,074ng/mL | √ | √ |
| Macrophage Inflammatory Protein-2  (MIP-2) | *Cxcl2* | 20310 | C; IR; Inf R | **1** | 7,2pg/mL | √ | √ |
| Macrophage Inflammatory Protein-3beta  (MIP-3β) | *Ccl19* | 24047 | C; IR; Inf R | **4** | 0,47ng/mL | × | √ |
| Matrix Metalloproteinase-9  (MMP-9) | *Mmp9* | 17395 | Collagen catabolic process | **1 (5)** | 10g/mL | √ | √ |
| Myeloperoxidase  (MPO ) | *Mpo* | 17523 | Oxidative stress | **1** | 0,95ng/mL | √ | √ |
| Myoglobin | *Mb* | 17189 | Enucleate erythrocyte differentiation | **1** | 24ng/mL | × | √ |
| Oncostatin M (OSM) | *Osm* | 18413 | MAPKKK; Apoptosis | **1, 2, 4** | 0,13ng/mL | × | √ |
| RANTES  regulated upon activation, normal T cell expressed and secreted | *Ccl5* | 20304 | C; IR; Inf R | **4** | 48pg/mL | × | × |
| Serum Amyloid P  (SAP) | *Apcs* | 20219 | Response to protein stimulus | **2** | 5,4ug/mL | × | √ |
| Stem Cell Factor  (SCF) | *Kitl* | 17311 | Negative regulation of apoptosis; MAPKKK cascade; Stem cell | **3** | 75pg/mL | × | √ |
| Serum Glutamic-Oxaloacetic Transaminase  (SGOT ) | *Got1* | 14718 | Amino acid metabolism | **1** | 1,9ug/mL | × | √ |
| Tissue Inhibitor of Metalloproteinase Type-1  (TIMP-1) | *Timp1* | 21857 | [erythrocyte maturation](http://www.informatics.jax.org/searches/GO.cgi?id=GO:0043249); [metalloendopeptidase inhibitor activity](http://www.informatics.jax.org/searches/GO.cgi?id=GO:0008191) | **1 (5)** | 0,18ng/mL | × | √ |
| Coagulation factor III  (Tissue Factor/TF) | *F3* | 14066 | Blood coagulation | **1 (5)** | 0,52ng/mL | √ | √ |
| Tumor Necrosis Factor-alpha (TNF-α) | *Tnf* | 21926 | Defense response; IR; Inf R; JNK cascade; leucocyte migration | **1, 2, 3, 4** | 0,14ng/mL | √ | √ |
| Thrombopoietin  (TPO ) | *Thpo* | 21832 | Cell Proliferation | **1** | 2,7ng/mL | √ | √ |
| Vascular Cell Adhesion Molecule-1  (VCAM-1) | *Vcam1* | 22329 | Leucocyte adhesion | **1** | 19ng/mL | × | √ |
| Vascular Endothelial Cell Growth Factor  (VEGF) | *Vegfa* | 22339 | Angiogenesis; anti apoptosis; blood vessel development; lung development | **1 (5)** | 38 pg/mL | √ | √ |
| von Willebrand Factor  (vWF) | *Vwf* | 22371 | Blood coagulation | **1** | 99 ng/mL | × | × |
| Heme oxygenase (decycling) 1 | *Ho1* | 15368 | Oxidative stress | **1** | ELISA | × | √ |
| Secreted phosphoprotein 1; Osteopontin | *Spp1* | 20750 | Antiapoptosis | **2, 4** | ELISA | × | √ |
| lipocalin 2 | *Lcn2* | 16819 | Response to virus transport | **1** | ELISA | × | √ |
| Fibroblast growth factor -basic | *Fgf2* | 14173 | MAPKKK, Angiogenesis, Lung development, apoptosis | **(5)** | 0,58ng/mL | √ | √ |

Immune response (IR); Chemotaxis (C); Inflammatory response (Inf R)
